# A Convenient Route to 4-Carboxy-4-Anilidopiperidine Esters and Acids 

**DOI:** 10.3390/molecules17032823

**Published:** 2012-03-07

**Authors:** János Marton, Brita Glaenzel, Julia Roessler, Daniela Golaszewski, Gjermund Henriksen

**Affiliations:** 1 ABX Advanced Biochemical Compounds, Biomedizinische Forschungsreagenzien GmbH, Heinrich-Glaeser-Strasse 10-14, D-01454 Radeberg, Germany; Email: marton@abx.de (J.M.); glaenzel@abx.de (B.G.); julia.roessler@chemie.tu-dresden.de (J.R.); golaszewski@abx.de (D.G.); 2 Department of Nuclear Medicine, Klinikum rechts der Isar, Technische Universität München, Ismaninger Strasse 22, D-81675 Munich, Germany

**Keywords:** 4-anilidopiperidines, *tert*-butyl ester, opioid receptors, positron emission tomography

## Abstract

The route selection and development of a convenient synthesis of 4-carboxy-4-anilidopiperidines is described. Previous routes were hampered by the low yield of the target esters as well as the inability to convert the esters to the required free acids. Considerations for large-scale production led to a modified synthesis that utilised a *tert*-butyl ester of 4-carboxy-4-anilidopiperidines which resulted in a dramatic increase in the overall yield of the target *N*-propionylated- 4-anilidopiperidine-4-carboxylic acids and their corresponding methyl esters. These compounds are now available for use as precursors and reference standards, of particular value for the production of ^11^C and ^18^F-labelled 4-carboxy-4-anilidopiperidine radiotracers.

## 1. Introduction

**Scheme 1:**
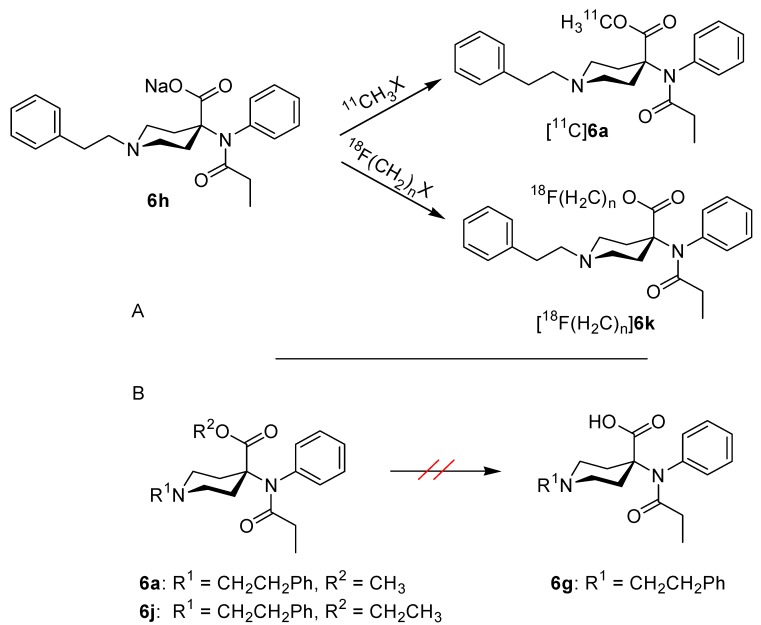
A: Synthesis of ^11^C-labelled carfentanil and ^18^F-derivatives. B: Failure to cleave simple alkyl esters of 4-AP-carboxylic acid.

The 4-anilidopiperidine (4-AP) [1,2] carfentanil (**6a**) is a highly potent μ-opioid-receptor (MOR) agonist [3,4]. [^11^C]**6a** (Scheme 1) is established for use as a tracer for MOR by means of positron emission tomography (PET) [5,6], whilst ^18^F-labelled derivatives with a potential for application in this non-invasive imaging technique [6] are in development (e.g., [^18^F(CH_2_)_2_]**6k** [7,8]). The synthesis of precursors for use in the radiosynthesis of [^11^C]**6a** and ^18^F-labelled analogues of **6a** (Scheme 1) relies on compound 4-[*N*-(1-oxopropyl)-*N*-phenylamino]-1-(2-phenylethyl)-4-piperidine-carboxylic acid (**6g**, desmethyl carfentanil free acid), its sodium [9,10,11] or ammonium [12,13] salt [7,8].

These approaches towards tracers for use in PET has so far been by the limited accessibility of acid **6g** by cleavage of **6a** or its ethyl ester analogue (**6j**, Scheme 1). Hydrolysis of the carboxylic acid esters in the carfentanil series (**6a**: R^2^ = CH_3_ or **6j**: R^2^ = CH_3_CH_2_) with commonly used reagents (e.g., KOH in ethylene glycol), results in the *N*-despropionyl compound (**4a**), presumably through an acyl-shift as was observed with the 4-AP sufentanil [14]. 

**Scheme 2:**
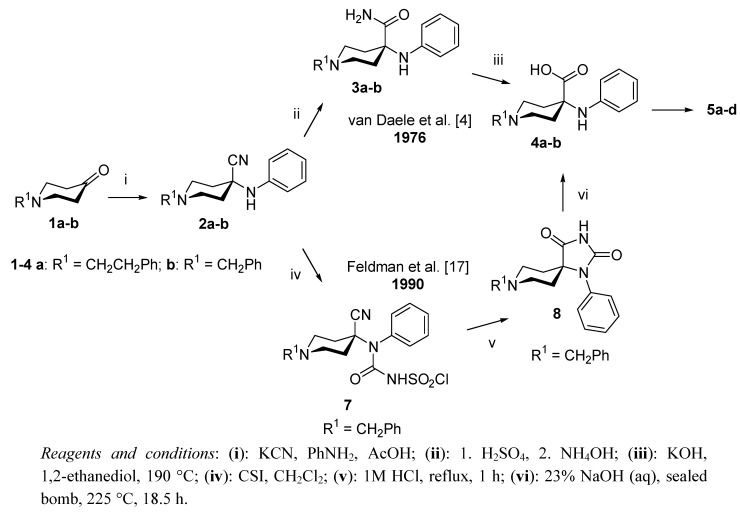
Preparation of 4-phenylamino-1-substituted-4-piperidine carboxylic acid derivatives.

**Scheme 3:**
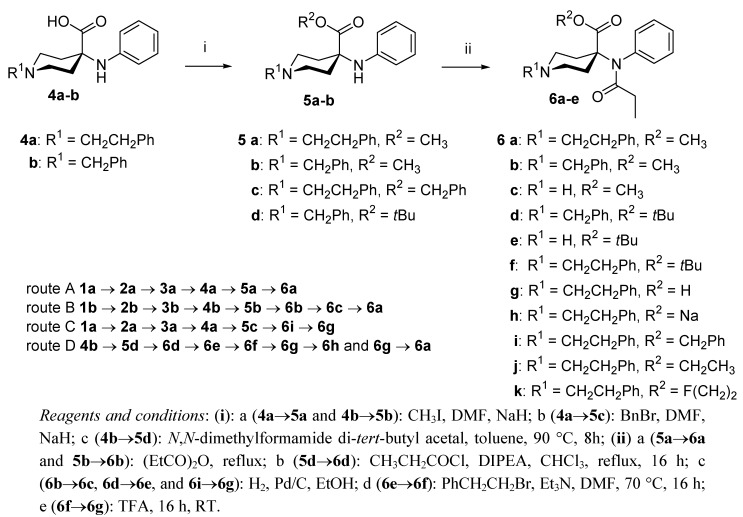
Synthesis of 4-carboxy-4-anilidopiperidine derivatives.

The original synthesis of **6a** is based on the preparation of α-phenylamino nitrile (**2a**) from 1-(2-phenylethyl)-4-piperidone (**1a**), aniline and KCN in a Strecker-addition (*route A: ***1a**→**2a**→**3a**→**4a**→**5a**→**6a**, Scheme 2 and Scheme 3). Nitrile hydrolysis yields carboxamide **3a**, which is finally reacted with KOH in 1,2-ethanediol at 190 °C to yield the free acid **4a**. Conversion to themethyl ester **5a** followed by acylation of **5a** with propionic anhydride results in **6a**. A limitation of this method is the very low overall yield (1.2% [3]) of **6a**, mainly caused by low conversion of the nitrile (**2a**) to the corresponding amide (**3a**, 14% [4], 3% [13]). Moreover, we found the modified reaction pathway [4] for the synthesis of **6a** not to provide any improvement over the original procedure (*route B*: **1b**→**2b**→**3b**→**4b**→**5b**→**6b**→**6c**→**6a**, 1.2% [15], Scheme 2 and Scheme 3) in contrast to the corresponding yield of 11% in the original report [4]. Furthermore, the preparation of **6g** according to procedures reported in the literature (*route C*: **1a**→**2a**→**3a**→**4a**→**5c**→**6i**→**6g**, Scheme 1 and Scheme 2) [4,8,12,13] also resulted in only a very low overall yield (0.5%). Finally, we identified more recently developed methods for the synthesis of **6a**, **6g** and **6h** [13,16] to be applicable only for reactions on the milligram scale.

## 2. Results and Discussion

The need for gram amounts of pure carfentanil acid for use as precursor in radiolabelling, as well as for the corresponding authentic reference compounds of the radiotracers in question, prompted us to address the development of an improved method of preparing desmethyl carfentanil free acid (**6g**), desmethyl carfentanil sodium salt (**6h**) and carfentanil (**6a**) itself. The literature procedures and the identified improved synthetic sequences are summarized in Scheme 2 and Scheme 3.

For the synthesis of **6a**, **6g**–**h**, compound **4b** was used as a key intermediate. **4b** was prepared [4,15] in a good overall yield (33%). An alternative method [17] for the preparation of **4b** was based on the reaction of α-aminonitrile **2b** with CSI, followed by cyclization of the resulting amide (**7**) by treatment with 1 M HCl to yield a 1-phenyl-spirohydantoin (**8**) derivate. Alkaline hydrolysis [18] of the 2,4-imidazilidinedione derivative yielded α-amino acid **4b** in an overall yield of 39%.

For our new synthesis route (*route D*: **4b**→**5d**→**6d**→**6e**→**6f**→**6g**→**6h** and **6g**→**6a**, Scheme 2 and Scheme 3) a *tert*-butyl group was chosen for protecting the carboxylic acid function of **4b**, ultimately providing the new compounds **5d**, **6d**–**f**. This protecting group has several advantages: The introduction of *tert*-butyl goup in **4b** is readily performed, and the final cleavage of the *tert*-butyl ester, subsequent to the required transformations, can be performed under mild conditions.

For the introduction of the *tert*-butyl group, **4b** was reacted with *N*,*N*-dimethylformamide di-*tert*-butyl acetal [19] or, alternatively, with *tert*-butyl 2,2,2-trichloroacetamidate [20] to yield the *tert*-butyl ester **5d** (71%/43%). *N*-propionlyation of **5d** was initially attempted by refluxing the amine in neat propionic anhydride, but this procedure led to the removal of the *tert*-butyl group. In contrast propionyl chloride in the presence of Hünig-base yielded **6d** in 60% yield. Hydrogenolysis followed by *N*-alkylation led to the new *tert-*butylester of carfentanil (**6f**). Deprotection of **6f** with neat TFA at ambient temperature afforded the target compound **6g**. Overall yields from **4b **were: **6g** (16.8%); **6h** (13.8%); **6a** (13.2%). Overall yields starting from **1b** were as follows: **6g** (5.54%); **6h** (4.55%); **6a** 4.35% which compare favourably to that of the literature procedures: 0.4–0.5%.

## 3. Experimental

### 3.1. General

Starting materials and reagents were obtained from major commercial suppliers and were used without further purifications. Melting points were measured with a Büchi-535 instrument and the reported data are uncorrected. ^1^H-NMR and ^13^C-NMR spectra were obtained with a Bruker 500 spectrometer, and measurements were obtained at 20 °C in CDCl_3_, CD_3_OD and DMSO-d_6_. Column chromatography was performed on silica gel (Kieselgel 60 Merck 1.09385 (0.040–0.063 mm)). TLC was accomplished on Macherey-Nagel Alugram^®^ Sil G/UV_254_ 40 × 80 mm aluminum sheets [0.25 mm silica gel with fluorescent indicator] with the following eluent systems (each (^v^/_v_)): [A]: hexane-ethyl acetate 8:2; [B]: chloroform-methanol 9:1; [C]: ethylacetate-methanol 8:2. The spots were visualized with a 254 nm UV lamp or with 5% phosphomolybdic acid in ethanol. **Abbreviations:** Ph: phenyl; Bn: benzyl; CSI: chlorosulfonyl isocyanate; TBTA: *tert* butyl 2,2,2-trichloroacetamidate; TFA: trifluoroacetic acid; DIPEA: *N*-ethyldiisopropylamine (Hünig-base), Caf: carfentanil, tBu: *tert* butyl group; Bn: benzyl group; βPh: aromatic part of a 2-phenylethyl group.

### 3.2. Chemistry

tert*-Butyl 4-phenylamino-1-benzyl-4-piperidinecarboxylate* (**5d**): Method A: 4-Phenylamino-1-benzyl-4-piperidinecarboxylic acid (**4b**) (3.1 g, 10 mmol) was dissolved in dry toluene (30 mL) under an argon atmosphere. The mixture was heated to 90 °C and *N*,*N*-dimethyformamide di-*tert*-butyl acetal (9.7 mL, 40 mmol) was added drop-wise over 40 min. The mixture was refluxed (oil-bath 110 °C) for 8h. The product mixture was cooled to ambient temperature and thereafter toluene (30 mL) was added. The organic phase was washed with saturated NaHCO_3_ solution (2 × 100 mL) and brine (100 mL), dried (Na_2_SO_4_) and the solvent was evaporated *in vacuo*. The crude product was purified by column chromatography (silica gel, eluent: hexane-ethyl acetate 8:2 (^v^/_v_). The product was dried *in vacuo* (3 × 10^−1^ mbar) for 24 h. Yield: 2.6 g (71%); m.p. 126–127 °C. R_f_[A] = 0.18; R_f_[B] = 0.90; R_f_[C] = 0.88. ^1^H-NMR (CDCl_3_) δ = 7.22–7.32 (m, 5H, CH_2_Ph); 7.12 (t, 2H, NHPh); 6.72 (t, 1H, NHPh); 6.61 (d, 2H, NHPh); 3.78 (s, 1H, NH); 3.51 (s, 2H, CH_2_Ph); 2.57 (m, 2H, CH_2_CH_2_); 2.43 (m, 2H, CH_2_CH_2_); 2.21 (m, 2H, CH_2_CH_2_); 1.98 (m, 2H, CH_2_CH_2_); 1.34 (s, 9H, (CH_3_)_3_C). ^13^C-NMR (CDCl_3_) δ = 174.3 (COOtBu); 145.5 (NPh-C1); 138.4 (Bn-C1); 129.0 (NPh-C3,5); 128.9 (Bn-C2,6); 128.2 (Bn-C3,5); 127.0 (Bn-C4); 118.3 (NPh-C4); 115.5 (NPh-C2,6); 81.0 ((CH_3_)_3_C); 63.0 (PhCH_2_); 58.8 (C-4); 49.1 (C-2,6); 33.4 (C-3,5); 27.8 ((CH_3_)_3_C). HRMS (ESI) Calcd for C_23_H_30_N_2_O_2_, 366.4966; [M+H]^+^: 367.2385; Found, [M+H]^+^: 367.2554.

Method B: To a solution of **4b** (4.11 g, 13.24 mmol) in dry dichloromethane (26 mL) and dry tetrahydrofuran (6 mL) *tert* butyl-2,2,2-trichloroacetamidate (TBTA, 8.71 g, 39.86 mmol) was added under argon atmosphere. Boron trifluoride diethyletherate (0.18 mL, 1.45 mmol) was added carefully at 0 °C and then the reaction mixture was stirred for 72 h at ambient temperature. It was filtered and water (50 mL) was added to the filtrate. The pH of the solution was adjusted to 9 with NH_4_OH. The suspension was extracted with chloroform (3 × 50 mL). The combined organic phase was dried (Na_2_SO_4_), filtered and the solvent was evaporated under reduced pressure. The crude product was purified by column chromatography as above. Yield: 2.13 g (43%).

tert*-Butyl 4-[*N*-(1-oxopropyl)-*N*-phenylamino]-1-benzyl-4-piperidinecarboxylate* (**6d**): The starting material **5d** (3.32 g, 9.05 mmol) was dissolved in dry chloroform (115 mL) and *N*-ethyldiisopropylamine (7.9 mL, 46 mmol) was added to the solution at ambient temperature. To the resulting mixture, propionyl chloride (2.4 mL, 27.6 mmol) was added drop-wise, and the reaction mixture was refluxed for 8 h. The mixture was cooled to room temperature and poured into water (150 mL). The organic phase was separated and the inorganic phase was extracted with chloroform (3 × 150 mL). The combined organic phases were washed with brine (100 mL), dried (Na_2_SO_4_), filtered and evaporated under reduced pressure. The residue was chromatographed on silica gel (eluent 1: hexane-ethyl acetate 7:3 (^v^/_v_), eluent 2: hexane-ethyl acetate 1:1 (^v^/_v_)). Yield: 2.32 g (60%); yellowish oil; R_f_[A] = 0.13; R_f_[B] = 0.92; R_f_[C] = 0.84. ^1^H-NMR (CDCl_3_) δ = 7.18–7.41 (m, 10H, CH_2_Ph, NPh); 3.47 (s, 2H, CH_2_Ph); 2.56 (m, 2H, CH_2_CH_2_); 2.44 (m, 2H, CH_2_CH_2_); 2.25 (m, 2H, CH_2_CH_2_); 1.84 (q, *J* = 7.3 Hz, 2H, COCH_2_CH_3_); 1.59 (m, 2H, CH_2_CH_2_); 1.50 (s, 9H, (CH_3_)_3_C); 0.95 (t, *J* = 7.3 Hz, 3H, COCH_2_CH_3_). ^13^C-NMR (CDCl_3_) δ = 173.5 (COOtBu); 172.3 (COCH_2_CH_3_); 139.8 (NPh-C1); 138.1 (Bn-C1); 130.6 (NPh-C3,5); 129.1 (Bn-C2,6; Bn-C3,5); 128.4 (Bn-C4); 128.1 (NPh-C2,6); 126.9 (NPh-C4); 80.8 ((CH_3_)_3_C); 63.2 (PhCH_2_); 62.8 (C4); 49.6 (C-2,6); 33.7 (C-3,5); 29.0 (COCH_2_CH_3_); 28.0 ((CH_3_)_3_C); 9.4 (COCH_2_CH_3_). HRMS (ESI) Calcd for C_26_H_34_N_2_O_3_, 422.5598; [M+H]^+^: 423.2647; Found, [M+H]^+^: 423.2767.

tert*-Butyl 4-[*N*-(1-oxopropyl)-*N*-phenylamino]-4-piperidinecarboxylate* (**6e**): A solution of **6d** (4.95 g, 11.7 mmol) in ethanol (200 mL) was hydrogenolysed under heterogenous catalytic conditions (10% Pd/C (1.24 g); 8 bar) at ambient temperature in an autoclave. After 18 h, the catalyst was removed by filtration and the solvent was evaporated. The residue was suspended in water (300 mL) followed by alkalization with NH_4_OH. The suspension was extracted with chloroform (4 × 150 mL) and the combined organic phase were dried (Na_2_SO_4_), filtered, and concentrated *in vacuo*. The crude material was purified by silica gel column chromatography (eluent: chloroform-methanol 9:1 (^v^/_v_)). The product was dried *in vacuo* (3.5 × 10^−1^ mbar, 48 h). Yield: 2.87 g (73%); yellowish oil R_f_[A] = 0.10; R_f_[B] = 0.40; R_f_[C] = 0.16. ^1^H-NMR (CD_3_OD) δ = 7.35–7.52 (m, 5H, NPh); 2.95 (m, 2H, CH_2_CH_2_); 2.80 (m, 2H, CH_2_CH_2_); 2.22 (m, 2H, CH_2_CH_2_); 1.90 (q, *J* = 7.5 Hz, 2H, COCH_2_CH_3_); 1.53 (s, 9H, (CH_3_)_3_C); 1.50 (m, 2H, CH_2_CH_2_); 0.95 (t, *J* = 7.5 Hz, 3H, COCH_2_CH_3_). ^13^C-NMR (CD_3_OD) δ = 175.9 (COOtBu); 173.4 (COCH_2_CH_3_); 140.5 (NPh-C1); 131.6 (NPh-C3,5); 130.6 (NPh-C2,6); 130.1 (NPh-C4); 82.3 ((CH_3_)_3_C); 64.6 (C-4); 43.2 (C-2,6); 34.8 (C-3,5); 29.9 (COCH_2_CH_3_); 28.3 ((CH_3_)_3_C); 9.8 (COCH_2_CH_3_). HRMS (ESI) Calcd for C_19_H_28_N_2_O_3_, 332.4373; [M+H]^+^: 333.2178; Found, [M+H]^+^: 333.2322.

tert*-Butyl 4-[*N*-(1-oxopropyl)-*N*-phenylamino]-1-(2-phenylethyl)-4-piperidinecarboxylate* (**6f**): To a solution of **6e** (0.93 g, 2.81 mmol) in *N*,*N*-dimethylformamide (15 mL) under an argon atmosphere, triethylamine (0.7 mL, 5 mmol) was added and the mixture stirred for 15 min. 2-Phenylethylbomide (0.5 mL, 3.69 mmol) was added drop-wise to the solution and the reaction mixture was stirred at 70 °C for 24 h. The solvent was evaporated *in vacuo*. Water (50 mL) and NH_4_OH (5 mL) was added to the residue and the suspension extracted with chloroform (4 × 50 mL). The combined organic extracts were washed with water (30 mL) and brine (30 mL), dried (Na_2_SO_4_), filtered, and concentrated under reduced pressure. The crude product was purified by column chromatography on silica gel (eluent 1: hexane, eluent 2: hexane-ethyl acetate 1:1 (^v^/_v_)). The product was dried in vacuo (2 × 10^−1^ mbar, 12 h). Yield: 0.99 g (81%); colourless oil R_f_[A] = 0.18; R_f_[B] = 0.80; R_f_[C] = 0.81. ^1^H-NMR (DMSO-d6) δ = 7.21–7.51 (m, 7H, CH_2_CH_2_Ph, NPh); 7.14 (m, 3H, NPh); 2.61 (m, 2H, CH_2_CH_2_Ph); 2.55 (m, 2H, CH_2_CH_2_Ph); 2.39 (m, 2H, CH_2_CH_2_); 2.32 (m, 2H, CH_2_CH_2_); 2.07 (m, 2H, CH_2_CH_2_); 1.76 (q, *J* = 7.5 Hz, 2H, COCH_2_CH_3_); 1.47 (m, 2H, CH_2_CH_2_); 1.42 (s, 9H, (CH_3_)_3_C); 0.83 (t, *J* = 7.5 Hz, COCH_2_CH_3_). ^13^C-NMR (DMSO-d6) δ = 172.3 (COOtBu); 171.3 (COCH_2_CH_3_); 140.4 (NPh-C1); 139.4 (βPh-C1); 130.4 (NPh-C3,5); 129.3 (βPh-C3,5); 128.5 (βPh-C2,6 and βPh-C4); 128.1 (NPh-C2,6); 125.7 (NPhC4); 79.7 ((CH_3_)_3_C); 62.2 (C-4); 59.6 (CH_2_CH_2_Ph); 49.1 (C-2,6); 33.0 (CH_2_CH_2_Ph); 32.8 (C-3,5); 28.3 (COCH_2_CH_3_); 27.6 ((CH_3_)_3_C); 9.3 (COCH_2_CH_3_). HRMS (ESI) Calcd for C_27_H_36_N_2_O_3_, 436.5864; [M+H]^+^: 437.2804; Found, [M+H]^+^: 437.2887.

*4-[*N*-(1-oxopropyl)-*N*-phenylamino]-1-(2-phenylethyl)-4-piperidinecarboxylic acid* (**6g**, desmethyl carfentanil free acid, CAS RN: [186022-53-7]): **6f** (955 mg, 2.18 mmol) was stirred with TFA (10 mL) at room temperature for 16 h under an atmosphere of argon. The solvent was evaporated and the residue was dried in vacuo (2 × 10^−1^ mbar, 16 h). The crude product was dissolved in 1M NaOH solution (4 mL) and then water was added (10 mL). The solution was filtered and the pH of the filtrate was adjusted to 5 with 1M HCl solution (3.5 mL) and it was left overnight at 0–4 °C. The precipitate was filtered, washed with cold methanol (10 mL) and dried in vacuo (2 × 10^−1^ mbar). The free acid was purified by RP-HPLC (Phenomenex 100A, Nucleosil C18; flow: 5 mL/min; mobil phase: methanol-water-acetic acid 70:30:0.1 (v/v/v), λ = 254 nm, t_R_ = 17.6 min). The product was dried in vacuo at room temperature (2 × 10^−1^ mbar, 16 h). Yield: 563 mg (67%); m.p. 230.0–233.5 °C. R_f_[A] = 0.04; R_f_[B] = 0.07; R_f_[C] = 0.06. ^1^H-NMR (DMSO-d6) δ = 12.43 (brs, 1H, COOH); 7.19–7.49 (m, 7H, CH_2_CH_2_Ph, NPh); 7.14 (m, 3H, NPh); 2.59–2.64 (m, 2H, CH_2_CH_2_Ph); 2.52–2.54 (m, 2H, CH_2_CH_2_Ph); 2.33–2.43 (m, 4H, 2 × CH_2_CH_2_); 2.08 (m, 2H, CH_2_CH_2_); 1.75 (q, *J* = 7.4 Hz, 2H, COCH_2_CH_3_); 1.52 (m, 2H, CH_2_CH_2_); 0.8 (t, *J* = 7.4 Hz, COCH_2_CH_3_). ^13^C-NMR (DMSO-d6 + CD_3_OD) δ = 174.2 (COOH); 173.9 (COCH_2_CH_3_); 139.0 (NPh-C1); 137.3 (βPh-C1); 130.9 (NPh-C3,5); 130.4 (βPh-C3,5); 129.9 (βPh-C2,6); 129.5 (βPh-C4); 129.4 (NPh-C4); 127.7 (NPh-C2,6); 60.2 (C-4); 57.2 (CH_2_CH_2_Ph); 49.8 (C-2,6); 30.9 (CH_2_CH_2_Ph); 30.3 (C-3,5); 29.1 (COCH_2_CH_3_); 9.7 (COCH_2_CH_3_). C_23_H_28_N_2_O_3_ (380.48).

*4-[*N*-(1-oxopropyl)-*N*-phenylamino]-1-(2-phenylethyl)-4-piperidinecarboxylic acid sodium salt* (**6h**, desmethyl carfentanil sodium salt, CAS RN: [98598-82-4]): The free acid **6g** (300 mg, 0.78 mmol) was dissolved in dry methanol (120 mL) at 60 °C and the solution was cooled to room temperature. Sodium methylate (43 mg) in dry methanol (10 mL) was given to the above solution and it was stirred at ambient temperature for 30 min. The solvent was removed by rotary evaporation and the residue was dried in vacuo (2 × 10^−1^ mbar, 72 h). Yield: 260 mg (82%); m.p. 114–116 °C. R_f_[A] = 0.06; R_f_[B] = 0.11; R_f_[C] = 0.10. ^1^H-NMR (CD_3_OD) δ = 7.38–7.48 (m, 5H, CH_2_CH_2_Ph); 7.23 (m, 2H, NPh); 7.14 (m, 3H, NPh); 2.69–2.75 (m, 4H, CH_2_CH_2_Ph and CH_2_CH_2_Ph); 2.67 (m, 2H, CH_2_CH_2_); 2.51 (m, 2H, CH_2_CH_2_); 2.33 (m, 2H, CH_2_CH_2_); 1.88 (q, *J* = 7.4 Hz, 2H, COCH_2_CH_3_); 1.07 (m, 2H, CH_2_CH_2_); 0.92 (t, *J* = 7.4 Hz, 3H, COCH_2_CH_3_). ^13^C-NMR (CD_3_OD) δ = 180.1 (COONa); 175.6 (COCH_2_CH_3_); 141.9 (NPh-C1); 141.3 (βPh-C1); 131.9 (NPh-C3,5); 130.1 (βPh-C3,5); 129.6 (βPh-C2,6); 129.4 (NPh-C2,6); 129.4 (βPh-C4); 127.0 (NPh-C4); 66.2 (C-4); 61.5 (CH_2_CH_2_Ph); 51.6 (C-2,6); 34.5 (CH_2_CH_2_Ph); 34.0 (C-3,5); 30.4 (COCH_2_CH_3_); 9.8 (COCH_2_CH_3_). C_23_H_27_N_2_NaO_3_ (402.46).

*Methyl 4-[*N*-(1-oxopropyl)-*N*-phenylamino]-1-(2-phenylethyl)-4-piperidinecarboxylate* (**6a**, carfentanil free base, CAS RN base: [59708-52-0]; Carfentanil oxalate salt: [61086-44-0]): **6g** (1.2 g, 2.87 mmol) was dissolved in dry methanol (20 mL) and refluxed in the presence of cc. sulfuric acid (0.4 mL) for 21 h under argon atmosphere. The solution was taken to room temperature and the solvent removed under reduced pressure. Water (25 mL) was added to the residue, and pH of the mixture adjusted to 9 with NH_4_OH. The suspension was extracted with a mixture of chloroform-methanol 5:2 (^v^/_v_). The organic phase was dried (Na_2_SO_4_) and the solvent evaporated. The resulting residue was dissolved in methylisobutylketone (20 mL) and oxalic acid dihydrate (0.38 g, 3 mmol) in methylisobutylketone (15 mL) was added. The white crystalls were filtered and dried in vacuo (3 × 10^−1^ mbar, 16 h). Yield: 1.1 g (79%); m.p. 183–184 °C. (Lit m.p. 189.5 °C [4], 188–189 °C [21], 182–184 °C [22]) ^1^H-NMR (DMSO-d6) δ = 7.15–7.50 (m, 10H, CH_2_CH_2_Ph, NPh); 3.67 (s, 3H, COOCH_3_); 3.24 (m, 2H, CH_2_CH_2_); 3.05 (m, 2H, CH_2_CH_2_Ph); 2.97 (m, 2H, CH_2_CH_2_); 2.83 (m, 2H, CH_2_CH_2_Ph); 2.23 (m, 2H, CH_2_CH_2_); 1.81 (m, 2H, CH_2_CH_2_); 1.77 (q, *J* = 7.4 Hz, 2H, COCH_2_CH_3_); 0.79 (t, *J* = 7.4 Hz, 3H, COCH_2_CH_3_). ^13^C-NMR (CDCl_3_) δ = 173.2 (COOCH_3_); 172.4 (COCH_2_CH_3_); 164.1 ((COOH)_2_); 138.4 (NPh-C1); 137.4 (βPh-C1); 130.3 (NPh-C3,5); 129.6 (βPh-C3,5); 129.0 (βPh-C2,6); 128.6 (βPh-C4); 128.5 (NPh-C2,6); 126.6 (NPh-C4); 60.1 (C-4); 56.6 (COOCH_3_); 52.3 (CH_2_CH_2_Ph); 48.6 (C-2,6); 30.3 (CH_2_CH_2_Ph); 30.0 (C-3,5); 28.2 (COCH_2_CH_3_); 9.0 (COCH_2_CH_3_). C_24_H_30_N_2_O_3_ (394.51). oxalate salt: C_26_H_34_N_2_O_7_ (484.54).

## 4. Conclusions

A simple and effective synthesis of 4-carboxy-4-anilidopiperidines has been developed based on coverting **4b** to the corresponding *t*-Bu ester for use as a key intermediate. The improved method facilitates the production of 4-carboxy-4-APs in general, and more specifically, opens a route for preparation of carboxy-4-APs for use in PET imaging. 

## Supplementary Materials

Supplementary materials can be accessed at: http://www.mdpi.com/1420-3049/17/3/2823/s1.
