# Supplementary material for: A Convenient Route to 4-Carboxy-4-Anilidopiperidine Esters and Acids
Source: Molecules. 2012 Mar 7;17(3):2823–32. doi: 10.3390/molecules17032823 (PMC6268299; doi:10.3390/molecules17032823)

Molecules **2012**, *17*, 2823-2832; doi:10.3390/molecules17032823

OPEN ACCESS

molecules

ISSN 1420-3049

www.mdpi.com/journal/molecules

Article

A Convenient Route to 4-Carboxy-4-Anilidopiperidine Esters and Acids

János Marton [1], Brita Glaenzel [1], Julia Roessler [1,†], Daniela Golaszewski [1] and Gjermund Henriksen [2,‡,*]

[1] ABX Advanced Biochemical Compounds, Biomedizinische Forschungsreagenzien GmbH, Heinrich-Glaeser-Strasse 10-14, D-01454 Radeberg, Germany; E-Mails: marton@abx.de (J.M.); glaenzel@abx.de (B.G.); julia.roessler@chemie.tu-dresden.de (J.R.); golaszewski@abx.de (D.G.)

[2] Department of Nuclear Medicine, Klinikum rechts der Isar, Technische Universität München, Ismaninger Strasse 22, D-81675 Munich, Germany

† Present Address: Department of Chemistry and Food Chemistry, Organic Chemistry I, Dresden University of Technology, Bergstrasse 66, D-01069 Dresden, Germany.

‡ Present Address: Scintomics Up North AS, Utveien 15, N-1430 As, Norway.

* Author to whom correspondence should be addressed; E-Mail: henriksen@scintomics.no; Tel.: +47-978-70-357.

Received: 3 January 2012; in revised form: 20 February 2012 / Accepted: 24 February 2012 / Published: 7 March 2012

Supporting Information

^{1}H-NMR spectrum of **5d** in $CDCl_3$	**2**
^{13}C-NMR spectrum of **5d** in $CDCl_3$	**3**
^{1}H-NMR spectrum of **6d** in $CDCl_3$	**4**
^{13}C-NMR spectrum of **6d** in $CDCl_3$	**5**
^{1}H-NMR spectrum of **6e** in CD_3OD	**6**
^{13}C-NMR spectrum of **6e** in CD_3OD	**7**
^{1}H-NMR spectrum of **6f** in DMSO-d6	**8**
^{13}C-NMR spectrum of **6f** in DMSO-d6	**9**
^{1}H-NMR spectrum of **6g** in DMSO-d6	**10**
^{13}C-NMR spectrum of **6g** in DMSO-d6 + CD_3OD	**11**
^{1}H-NMR spectrum of **6h** in CD_3OD	**12**
^{13}C-NMR spectrum of **6h** in CD_3OD	**13**
^{1}H-NMR spectrum of **6a** in DMSO-d6	**14**

^{1}H-NMR spectrum of **5d** in $CDCl_3$.

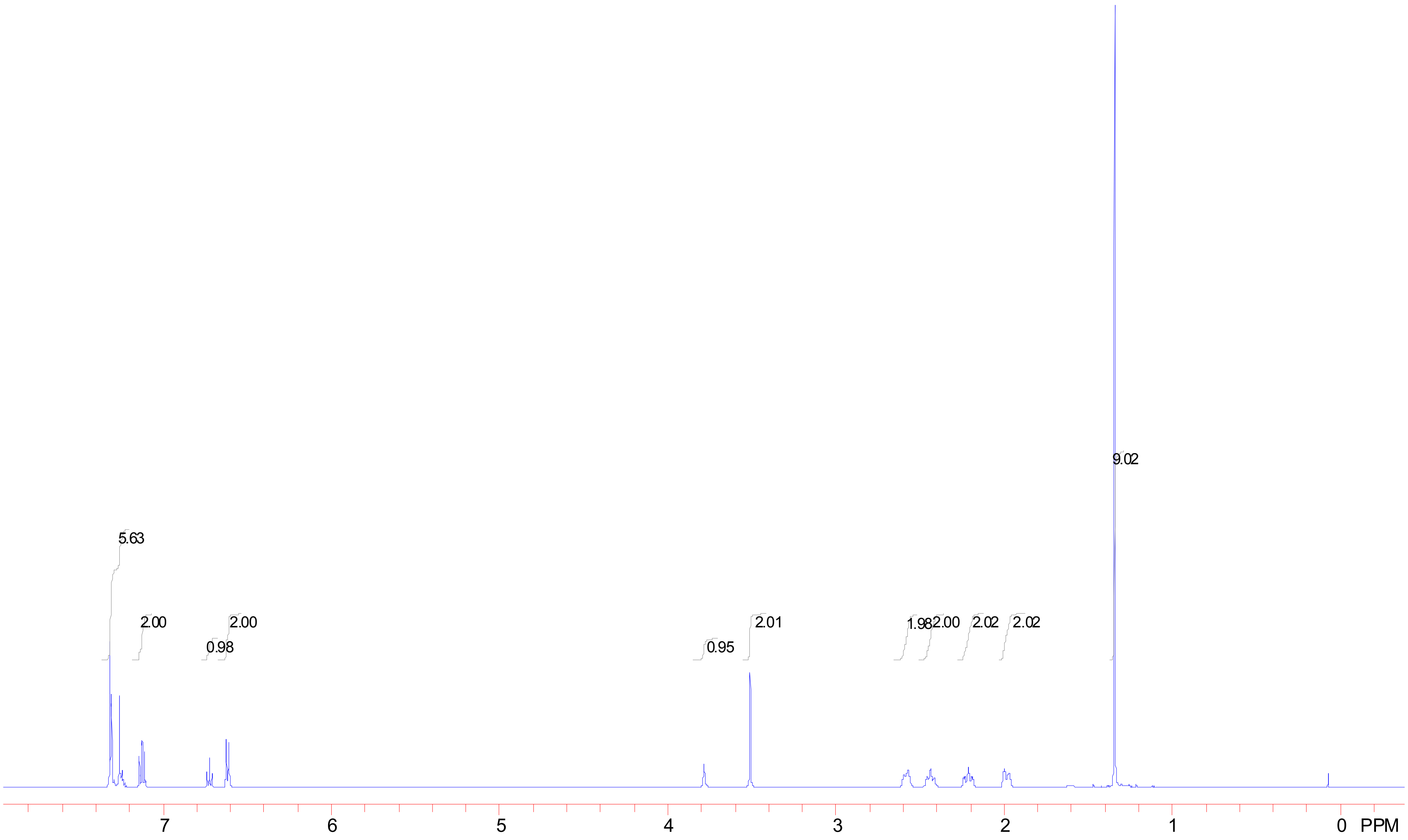

^{13}C-NMR spectrum of **5d** in $CDCl_3$.

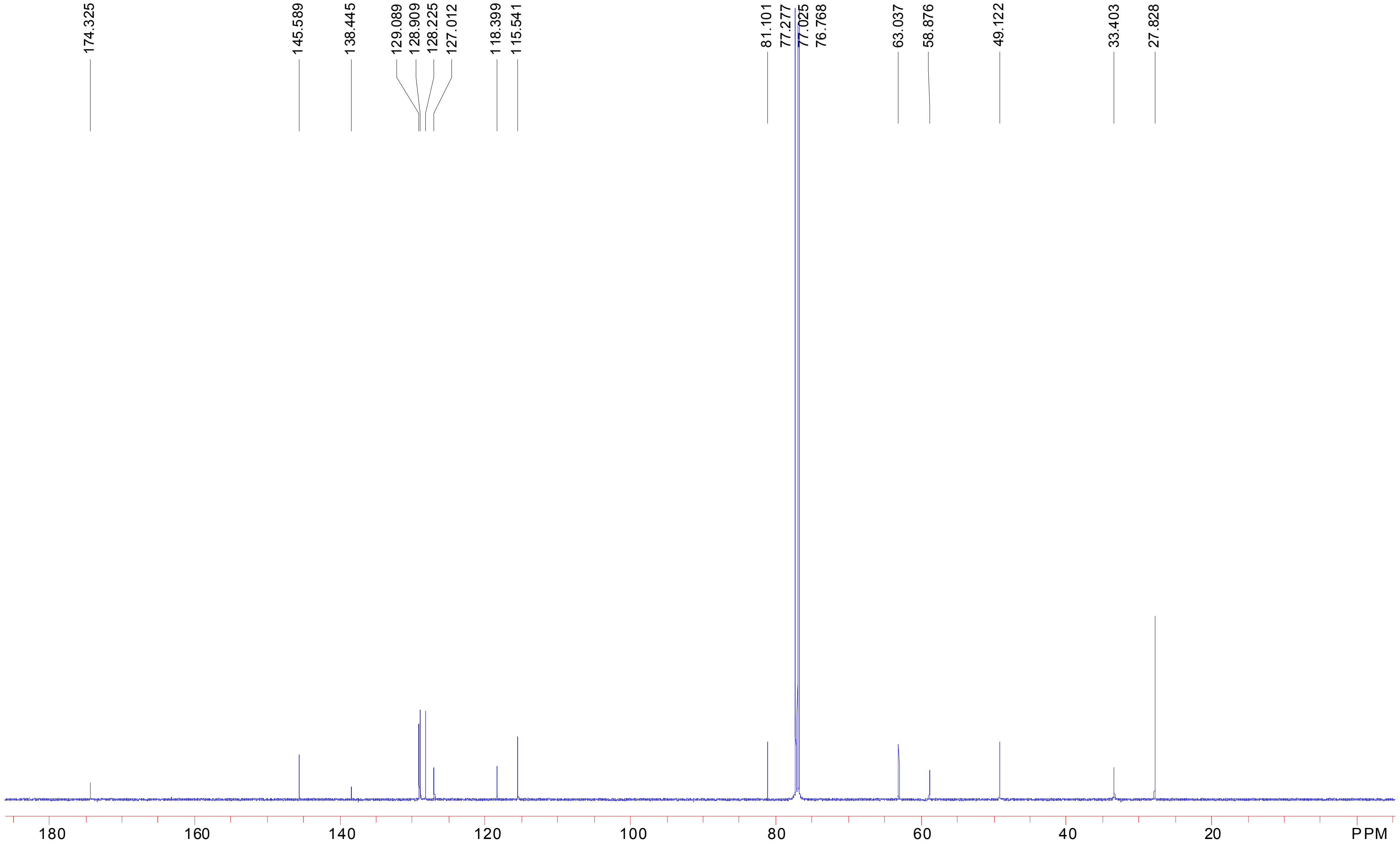

^{1}H-NMR spectrum of **6d** in $CDCl_3$.

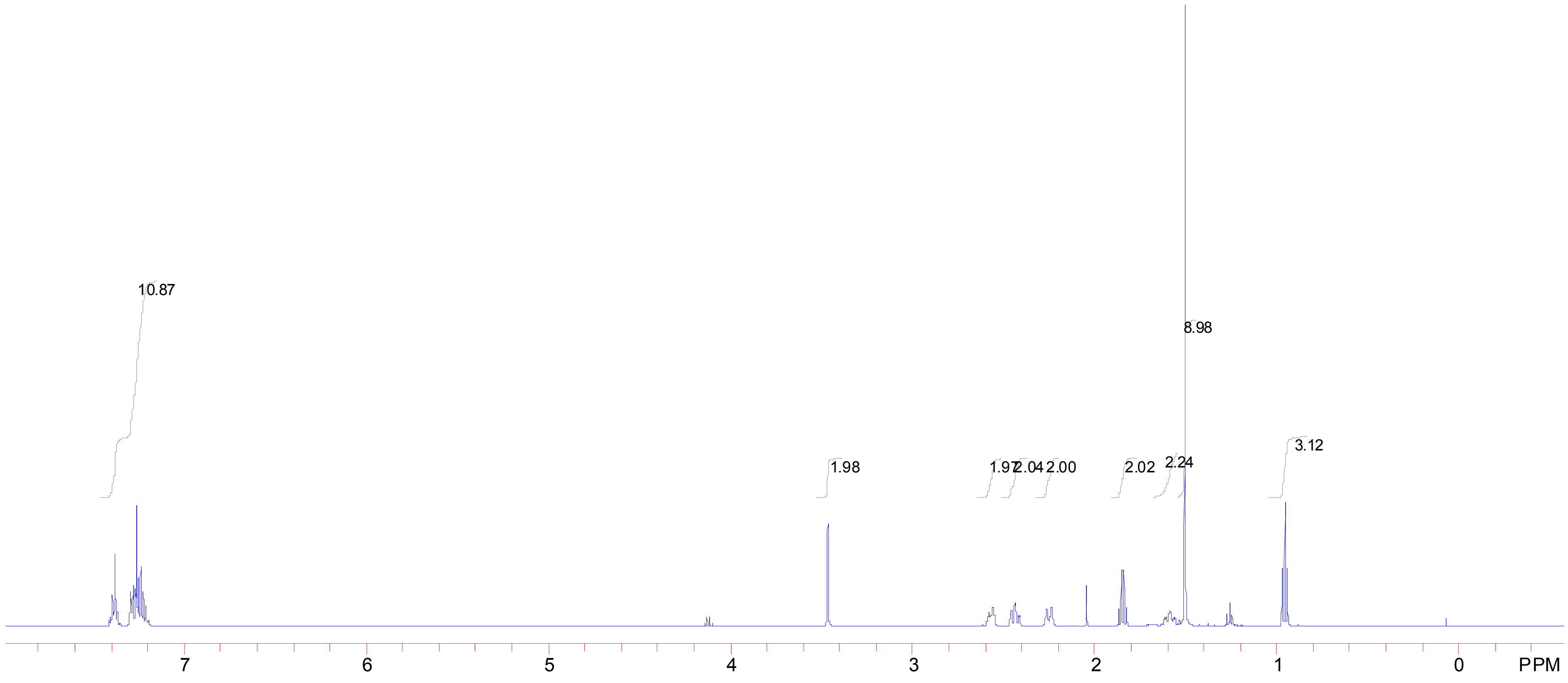

^{13}C-NMR spectrum of **6d** in $CDCl_3$.

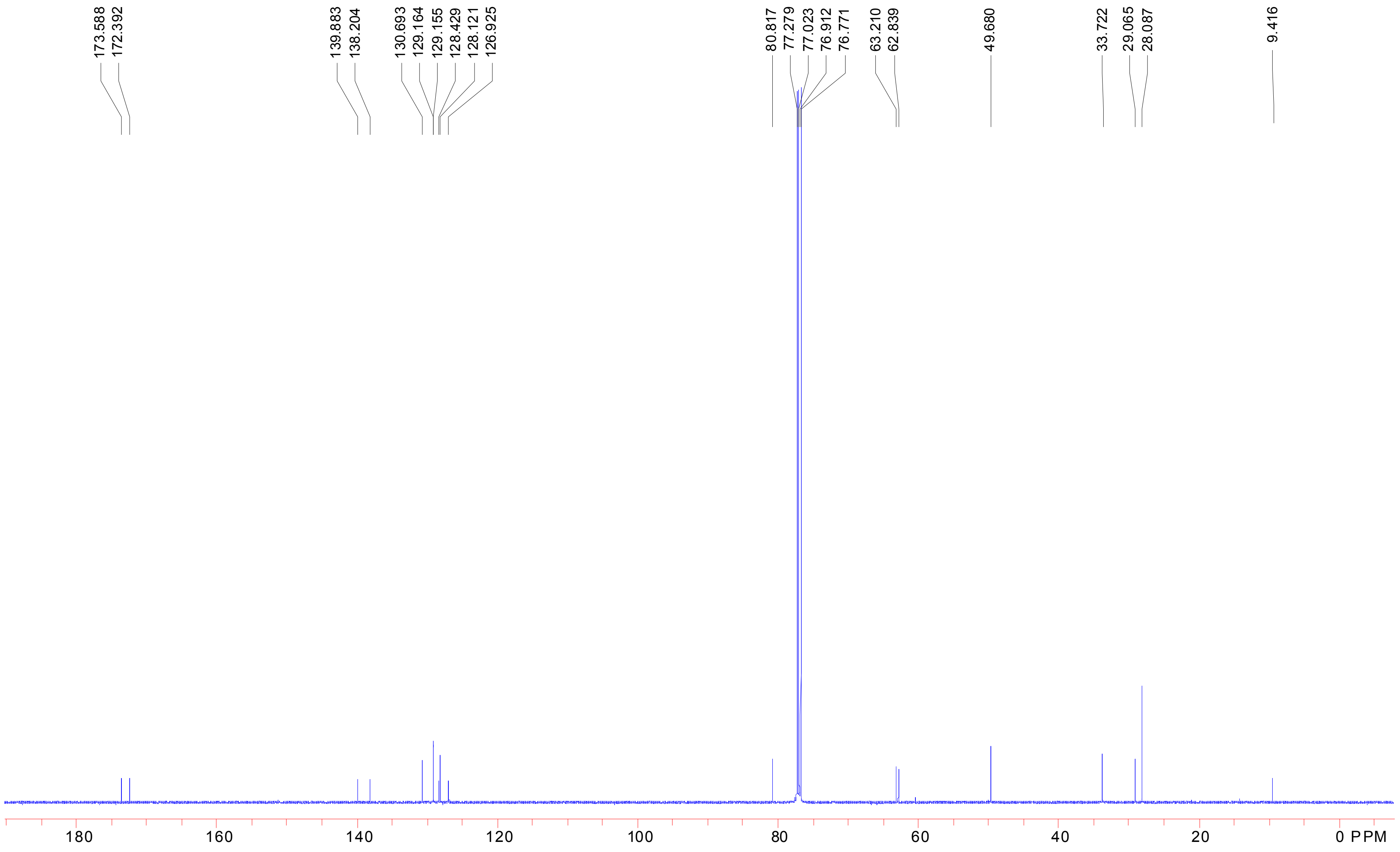

^{1}H-NMR spectrum of **6e** in CD_3OD.

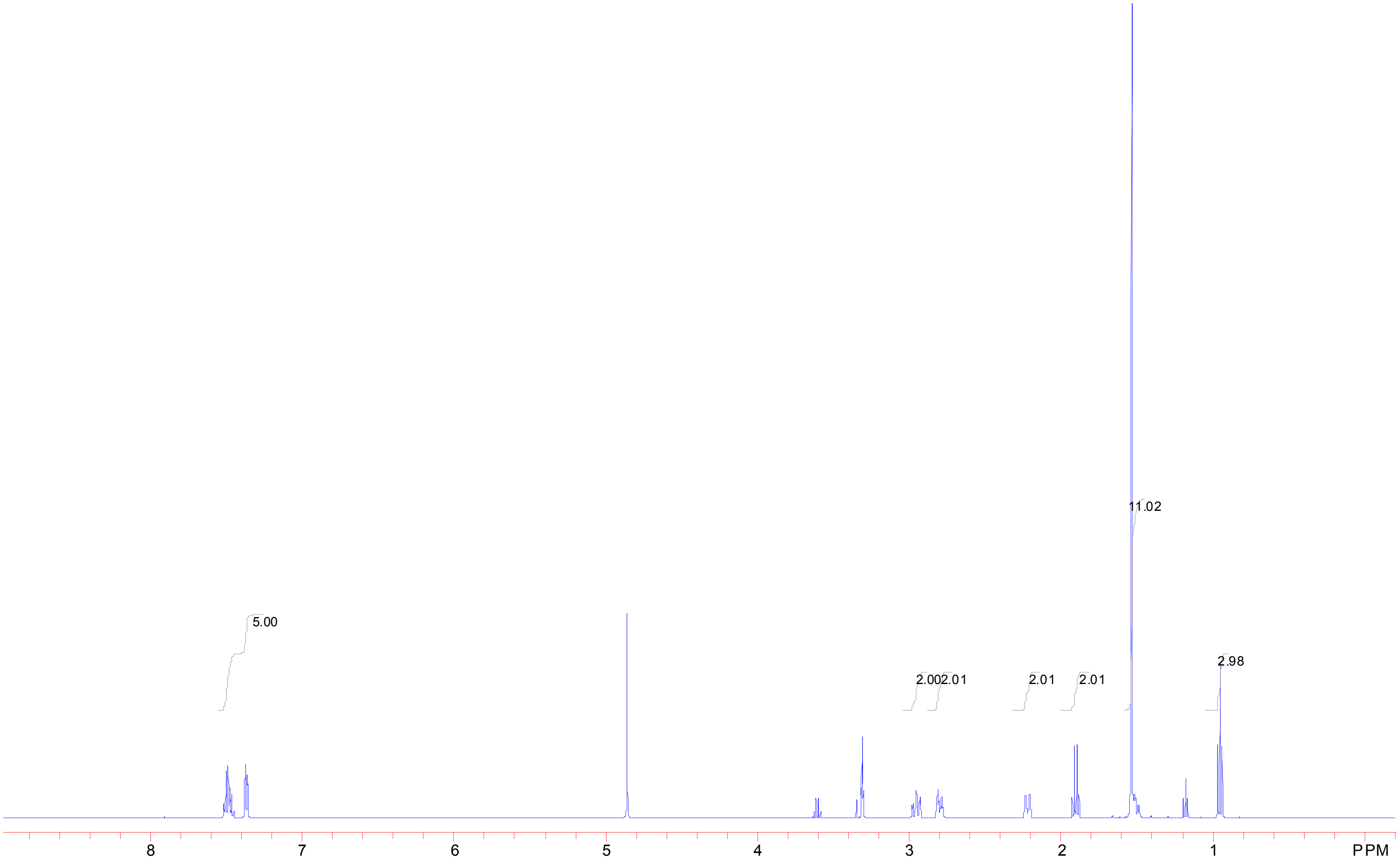

^{13}C-NMR spectrum of **6e** in CD_3OD.

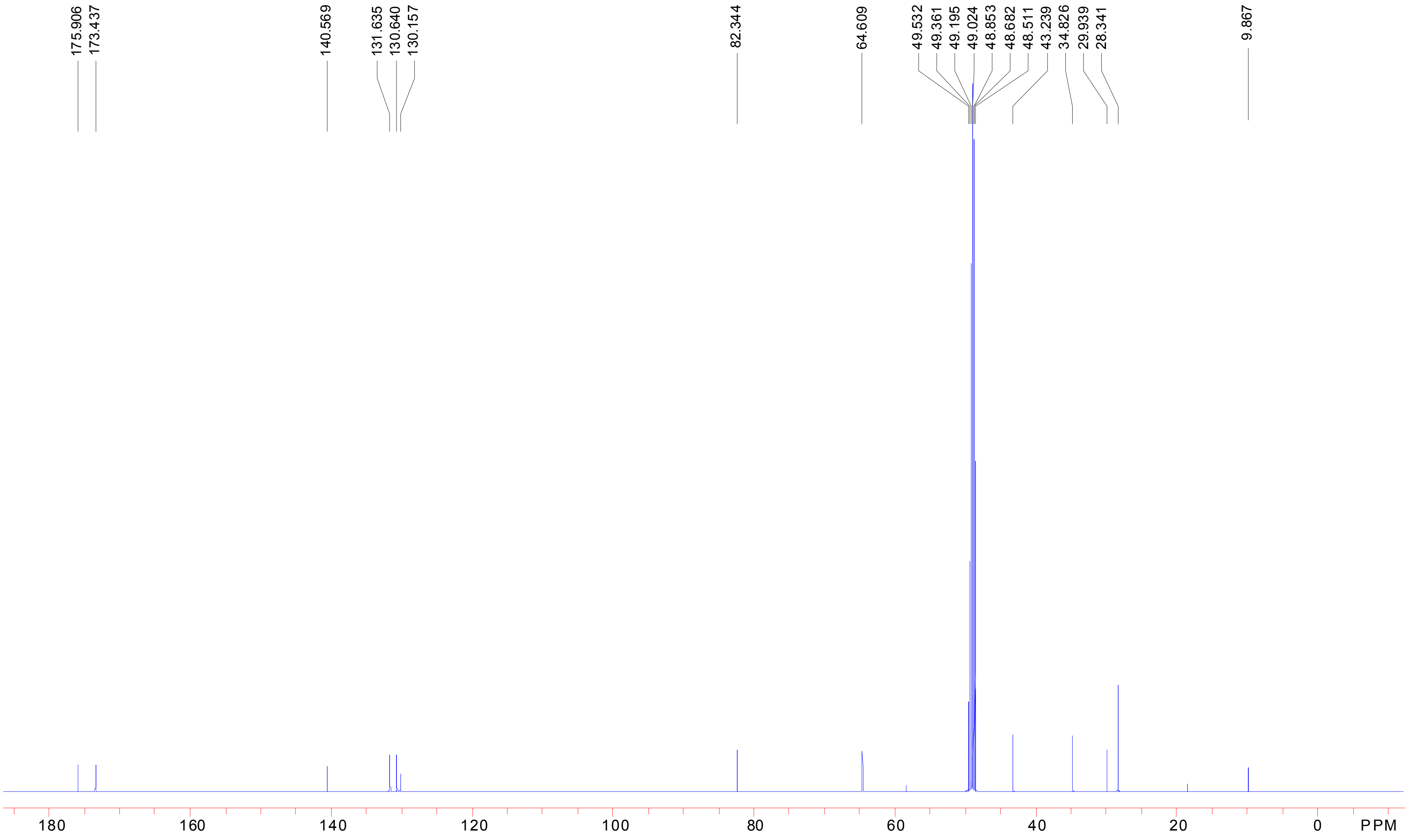

^{1}H-NMR spectrum of **6f** in DMSO-d6.

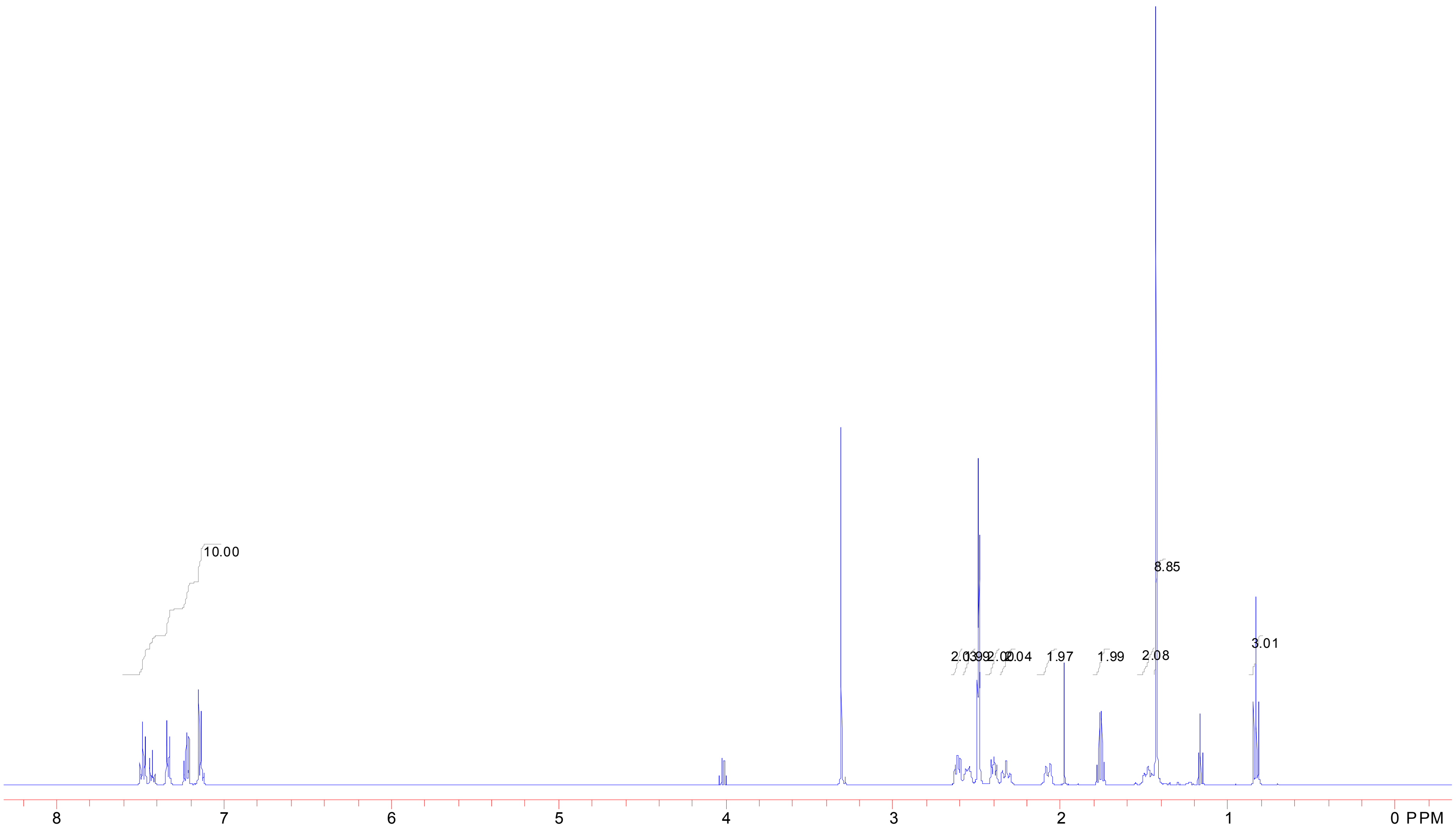

^{13}C-NMR spectrum of **6f** in DMSO-d6.

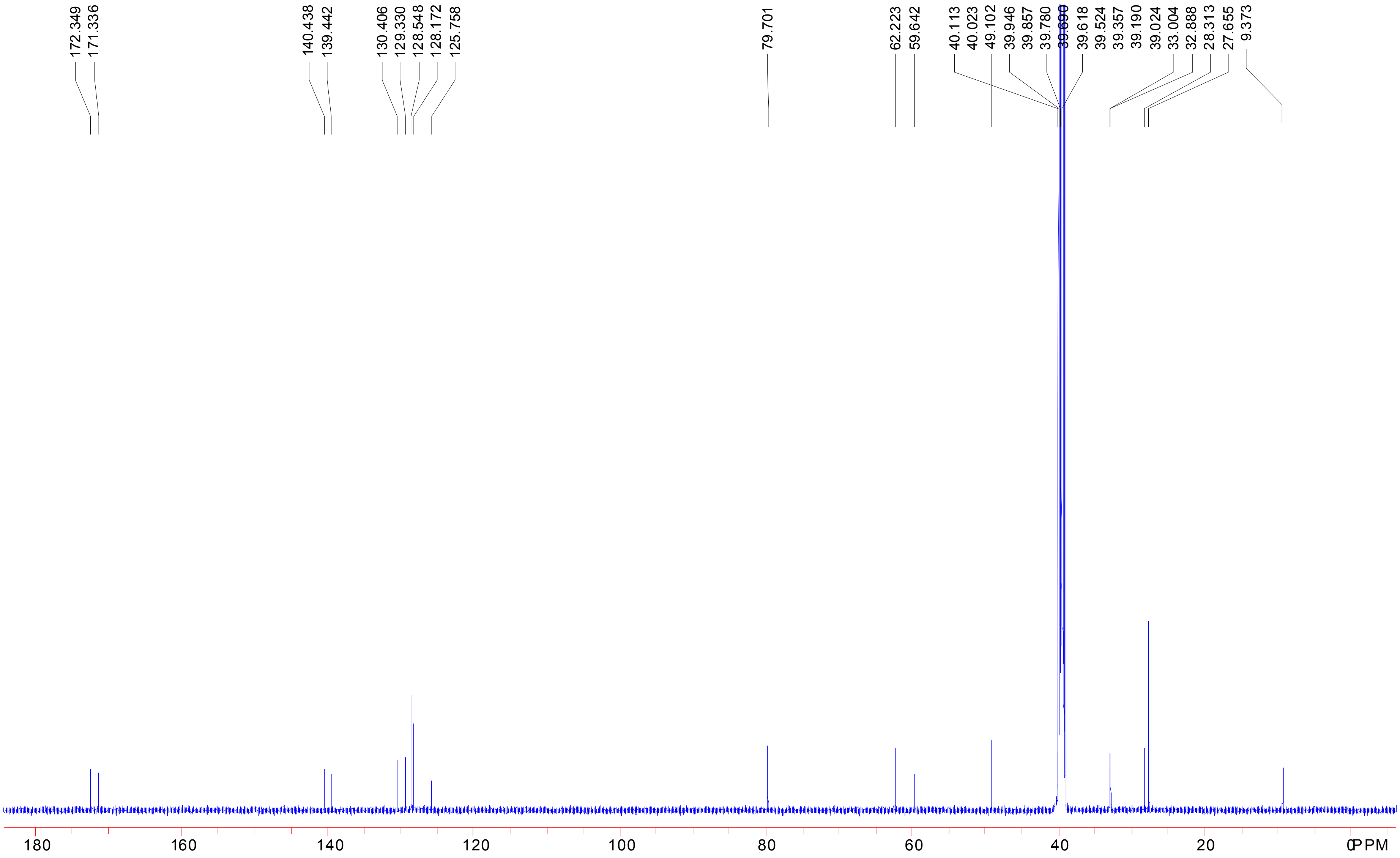

^{1}H-NMR spectrum of **6g** in DMSO-d6.

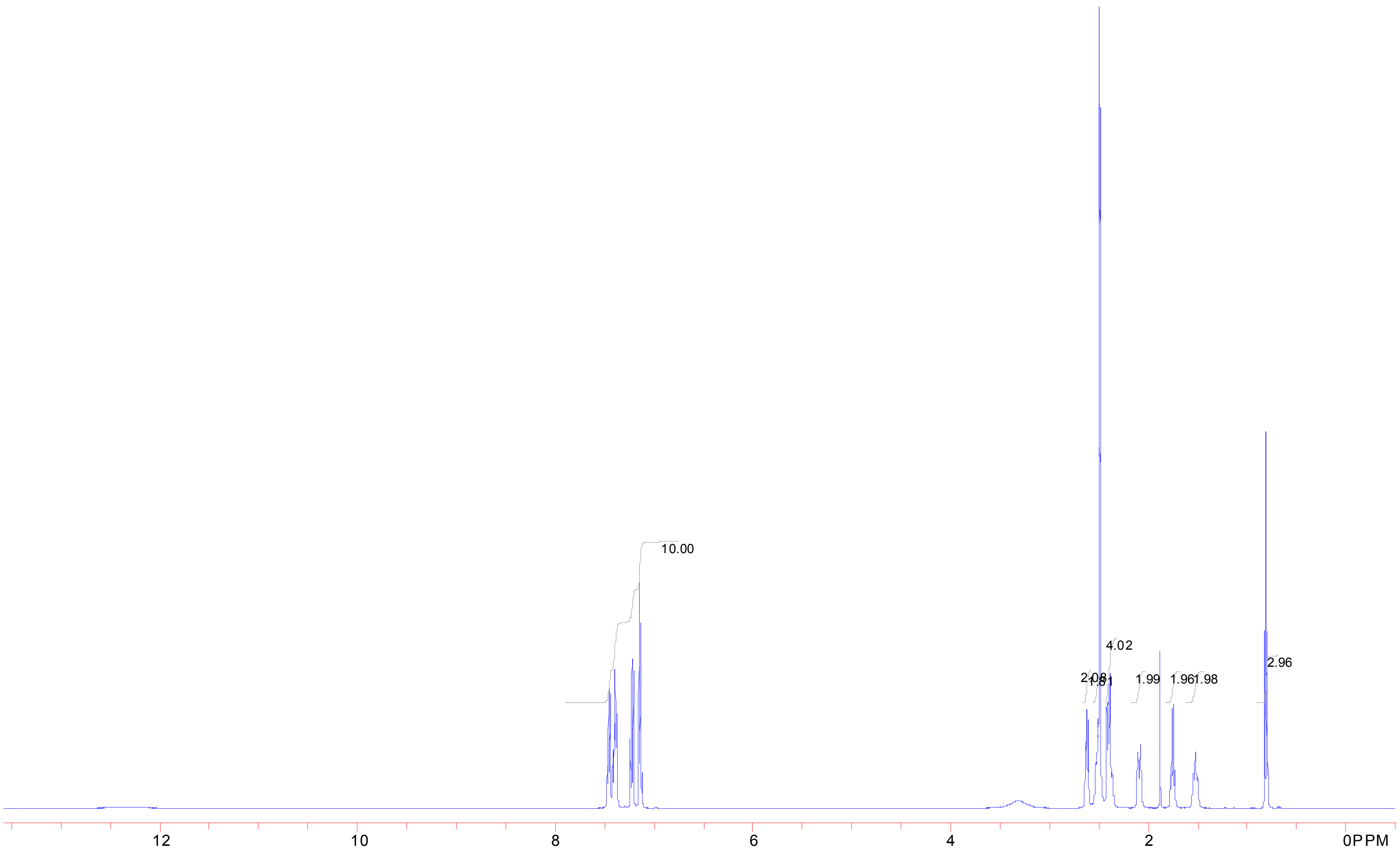

^{13}C-NMR spectrum of **6g** in (DMSO-d6 + CD_3OD).

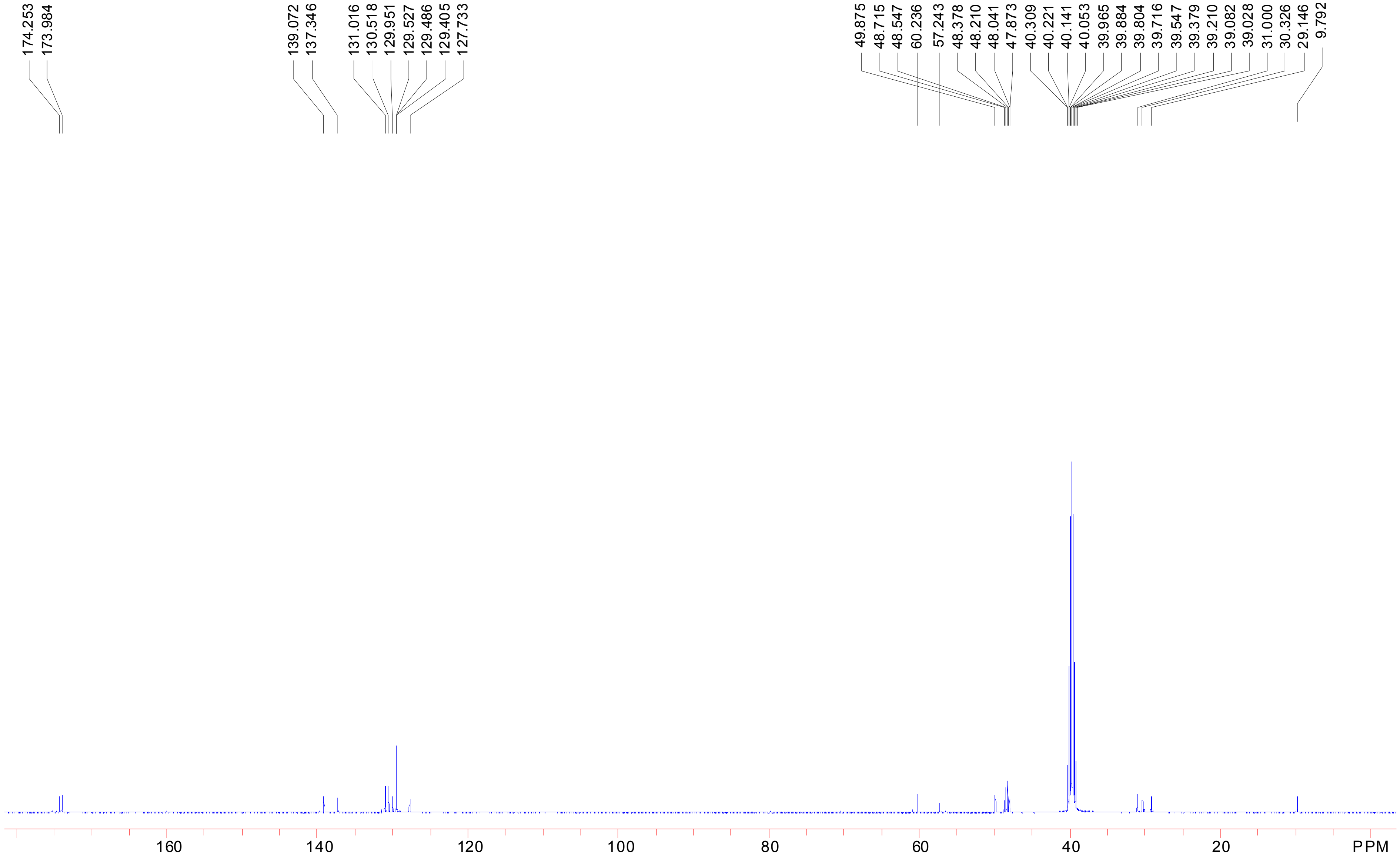

^{1}H-NMR spectrum of **6h** in CD_3OD

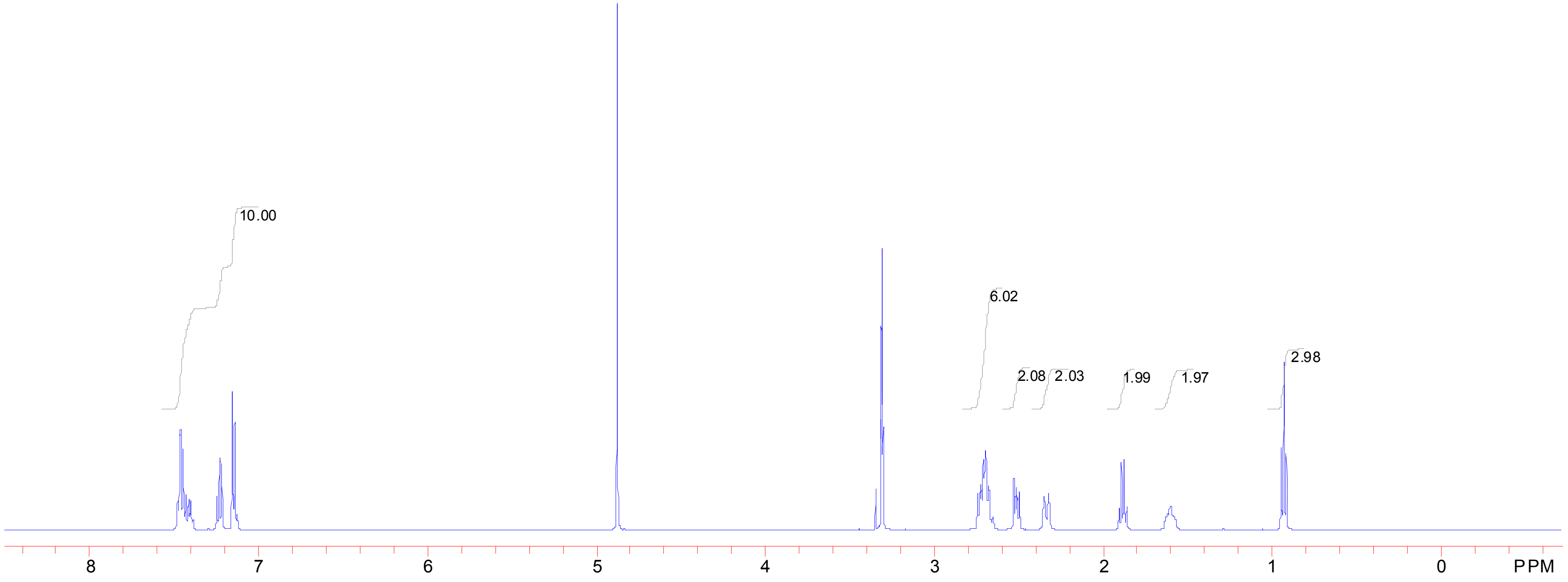

^{13}C-NMR spectrum of **6h** in CD_3OD.

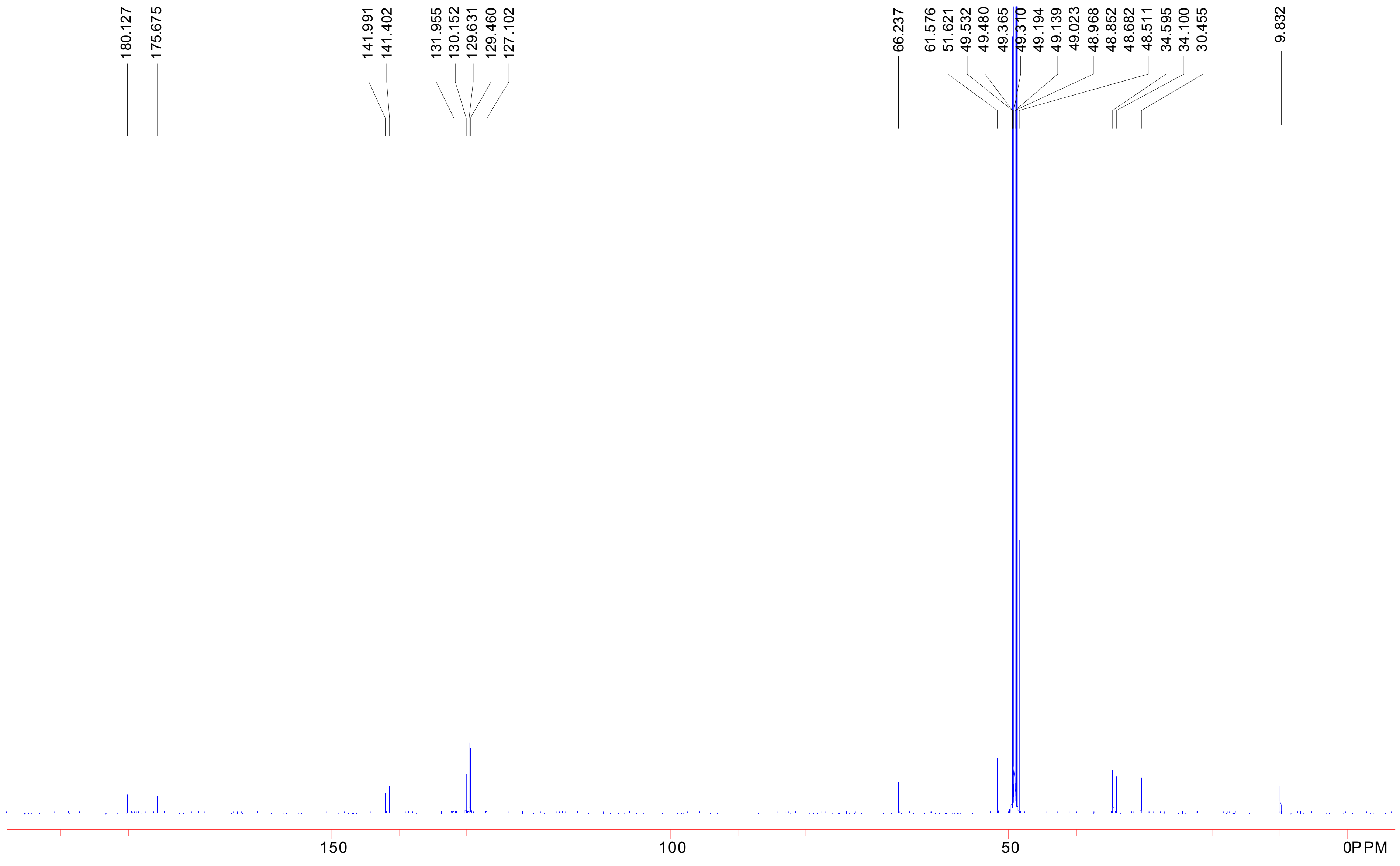

^{1}H-NMR spectrum of **6a** in DMSO-d6.

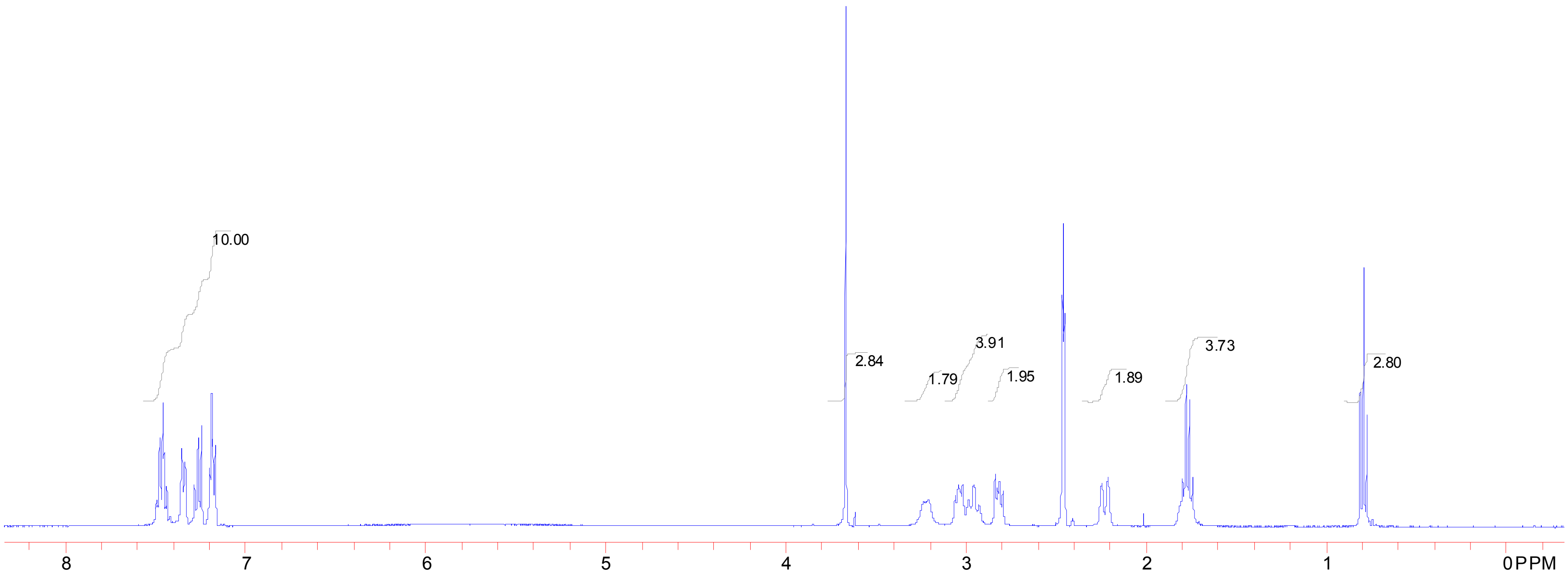

Supplement: Supplementary file 1 [file molecules-17-02823-s001.pdf]
